# Supplementary material for: Heterologous expression of genes from a cyanobacterial endosymbiont highlights substrate exchanges with its diatom host
Source: PNAS Nexus. 2023 Jun 27;2(6):pgad194. doi: 10.1093/pnasnexus/pgad194 (PMC10299089; doi:10.1093/pnasnexus/pgad194)
Supplement: pgad194_Supplementary_Data [file pgad194_supplementary_data.zip › PNASNEXUS-PNASNEXUS-2023-00139R-s03.pdf]

Suppl. Table S1. ***Richelia euintracellularis*** **ABC transporters studied in this work.** The ABC components of *Anabaena* sp. strain PCC7120 transporter used as query in BlastP analysis are shown.

Anabaena transporter (substrates)	ReuHH01 homologs (expect value in BlastP analysis)<br>SBP, periplasmic solute binding protein<br>TMD, transmembrane domain/protein<br>NBD, nucleotide binding domain (ATPase)				
Gls (glucosides)	RintHH_17430 (9e-21) SBP	RintHH_290 (8e-52) TMD	RintHH_15070 (9e-47) TMD	RintHH_1954 (1e-166) NBD	
N-I (neutral/ hydrophoic amino acids)	RintHH_11820 (0.0) SBP	RintHH_6690 (2e-85) NBD	RintHH_6700 (0.0) TMD	RintHH_12020 (e-158) TMD	RintHH_22020 (e-121) NBD
N-II (polar/acidic amino acids)	RintHH_12770 (e-166) SBP	RintHH_12760 (e-155) TMD	RintHH_12750 (e-163) TMD	RintHH_6220 (e-154) NBD	
Pot (spermidine/ polyamines)	RintHH_7180 (3e-26) SBP	RintHH_17090 (3e-13) TMD	RintHH_17600 (2e-79) NBD		

Table S2. Specific oligonucleotides designed to detect gene transcripts similar to ReuHH01 *invB*, *glsR*, *natF*, *potD* and *secA*. Ref Seq, reference sequence.

**Gene**	**Ref Seq**	**Forward Primer (5´to 3´)**	**Probe**	**Reverse Primer (5´to 3´)**	**Design tool**	**Gblock size (bp)**
secA	RintHH_16190	GTTGGAAGCAGAAGAGAAAG	TGCAGCAGAGAAAGCTCCAACTGA	CTGATAAGCATCTCGTAGTATTG	IDT Primer Quest™	780
glsR	RintHH_1740	GCTTCCAAAAAACCTGGATTACA	CAAATGACTGTAGCTCGTGCTCGGCC	CAGATAAACGACTATACCCAGCCATA	Primer Express™ v3.0	1320
invB	RintHH_3860	CGAGCAGCCCGGGAATTATTA	ATCGCCTACCCGTACTGGCCGCTC	GATCAATCCAGTAATGCTTGCGAATA	IDT Primer Quest™	1434
natF	RintHH_2770	TGCCAGAACGAGAAAATCATATTATC	TGAATCCCTTTCTCAAGAACCCCTTGCA	TTAAGGCATCAGCCCATTTTG	Primer Express™ v3.0	1047
potD	RintHH_7180	CGCAACCAGCAAGGTGATTTAG	TCGTCAGGGCCAGATTTGGGCAGCA	TGTGGTTCCCAACCTAATCTATCAA	NCBI Primer-BLAST	1143

Suppl Table S3: Summary of qRT-PCR analyses. Estimates of gene expression for target genes that encode for substrate binding proteins (*glsR, natF, potD*), an invertase (*invB*), and the housekeeping gene, *secA*. The following abbreviations apply: bd. below detectio; dnq: detected, not quantifiable; nr: not run; na: not applicable. DC indicate samples with higher values in the no-RT controls than in samples with amended cDNA template.

							**cDNA L^{-1} (standard error)**				
**Sample ID**	**Station**	**Dec Long**	**Dec Lat**	**Depth (m)**	**Vol (L)**	**Time (local)**	***glsR***	***invB***	***natF***	***potD***	***secA***
64	2	-54.514	10.288	2	0.75	13:20	9.88e+03 (1.65e+03)	1.31e+04 (1.19e+03)	1.09e+05 (1.16e+04)	3.91e+03 (6.61e+02)	1.42e+04 (4.92e+02)
65	2			10	0.5	13:20	4.80e+03 (2.43e+02)	7.19e+03 (6.80e+02)	6.11 e+04 (4.86e+02)	2.71e+03 (8.71e+02)	4.73e+03 (9.97e+02)
68	2			45	1	13:20	dnq (na)	DC (na)	nr (na)	nr (na)	nr (na)
87	5	-49.981	6.814	101	2.5	11:30	2.37e+02 (2.81e+01)	1.08e+02 (2.69e+01)	2.55e+02 (1.22e+01)	dnq (nd)	1.56e+02 (1.37e+01)
88	5			69	2.5	11:30	1.14e+02 (6.30e+01)	2.57e+02 (3.48e+01)	7.92e+02 (2.55e+01)	7.08e+01 (1.99e+01)	4.76e+02 (2.72e+01)
89	5			50	2.5	11:30	2.53e+02 (5.86e+01)	2.35e+02 (2.89e+01)	6.95e+02 (6.19e+01)	3.84e+02 (2.17e+02)	5.30e+02 (1.01e+01)
90	5			21	2.5	11:30	dnq (na)	dnq (na)	2.61e+02 (2.66e+01)	dnq (na)	2.43e+02 (1.06e+02)
91	5			10	2.5	12:49	bd (na)	bd (na)	bd (na)	bd (na)	bd (na)
92	5			4	1.25	12:50	nd (na)	nd (na)	nd (na)	nd (na)	nd (na)
93	5			4	1.25	23:15	dnq (na)	dnq (na)	2.97e+03 (4.21e+02)	4.26e+01 (2.40e+01)	2.24e+02 (1.96e+01)
94	5			10	1.25	23:15	2.28e+02 (1.12e+02)	2.72e+02 (1.44e+02)	2.07e+03 1.47e+02)	dnq (na)	2.71e+02 (8.61e+01)
95	5			21	1.25	23:15	1.05e+02 (9.21e+0)	dnq (na)	dnq (na)	7.10e+02 (2.11e+02)	nr (na)
96	5			45	1.25	23:15	DC (na)	DC (na)	nr (na)	nr (na)	nr (na)

							cDNA L^{-1} (standard error)				
Sample ID	Station	Dec Long	Dec Lat	Depth (m)	Vol (L)	Time (local)	*glsR*	invB	natF	potD	secA
260	23	-54.448	10.769	102	2.5	8:55	6.52e+02 (1.06e+02)	5.85e+02 (9.07e+01)	1.42e+03 (6.39e+01)	4.17e+02 (1.59e+02)	8.43e+02 (1.57e+02)
261	23			61	2.42	9:19	1.85e+03 (2.79e+01)	2.56e+03 (4.94e+02)	8.44e+03 (3.26e+02)	2.24E+03 (1.70e+02)	4.73E+03 (3.76e+02)
262	23			38	2.35	8:44	1.19e+03 (2.21e+02)	2.53e+03 (4.44e+02)	8.29E+03 (2.31e+02)	1.90E+03 (1.18e+02)	5.78E+03 (3.26e+02)
263	23			24	2.5	9:00	dnq (na)	dnq (na)	dnq (na)	nr (na)	bd (na)
264	23			10	2.5	10:10	1.42e+02 (7.01e+01)	1.14e+02 (3.85e+01)	5.58e+02 (4.89e+01)	5.58e+02 (1.23e+01)	2.51e+02 (1.39e+02)
265	23			4	2.5	10:00	dnq (na)	dnq (na)	7.63e+01 (2.05e+01)	nr (na)	nd (na)
281	25	-56.37	11.313	26	2.5	10:45	1.98e+03 (1.03e+02)	1.10e+03 (3.29e+02)	2.48e+03 (2.39e+02)	2.10e+03 (1.86e+02)	2.33e+03 (3.09e+02)
282	25			26	2.5	11:19	4.78e+03 (4.14e+02)	7.20e+03 (6.11e+02)	2.17e+04 (1.76e+02)	3.85e+03 (1.83e+02)	1.37e+04 (3.87e+02)
283	25			26	2.5	13:30	3.32e+02 (1.75e+01)	3.60e+02 (1.10e+02)	1.75e+03 (7.91e+01)	2.95e+02 (6.70e+01)	1.12e+03 (6.55e+01)
284	25			26	2.5	16:20	4.02e+03 (1.84e+02)	4.37e+03 (2.49e+02)	9.97e+03 (2.05e+02)	3.20e+03 (1.32e+02)	5.95e+03 (8.36e+01)
326	25			26	2.5	10:45	DC (na)	3.16e+02 (9.03e+01)	nr (na)	nr (na)	nr (na)
327	25			26	2.5	10:45	1.19e+03 (3.91e+02)	1.33e+03 (5.29e+02)	nr (na)	nr (na)	nr (na)

Suppl. Table S4. Oligodeoxynucleotide primers used in plasmid construction and PCR analysis. The sequences of the indicated restriction enzyme sites are underlined.

**Primer Name**	**Sequence (5´to 3´)**
pET28b_2F	CTTATGCGACTCCTGCATTAG
GlsR-1	CAGTGGTTGGGAATGCTC
GlsR-2	GGTTGGCCATCACCATTA
T7-terminator primer	GCTAGTTATTGCTCAGCGG
F7	GTATCATGGTTGTGGTGGGC
R1	CGGTAGGCGGTTATCAATTGCTTC
CK.3-5	CCTTAAAACATGCAGGAATTGACG
RintHH_2	CTACGTGCTTATGTTGTCGC
prL500-1	ATAGGCGTATCACGAGGC
RintHH_6	GACCCCATATGATGCAACCACATAAAGTAATTC
RintHH_7	GCCTGCTCGAGTTAAGAGTCAAAGTAGTCAAATTTTAAC
RintHH_12770-3 (NdeI)	AAAGCATATGTGTAGTAGTAATACAGTCAC
RintHH_12770-4 (XhoI)	CTCTCGAGTTACTATCTAAATGGTGGA
RintHH_7180-3 (NdeI)	AAGCATATGTGTAGCAGTAAGCAAAAGAC
RintHH_7180-4 (XhoI)	AGACTCGAGTTAAGCTTTGGTGATTCTA
RintHH_11820-3 (NcoI)	TAACCATGGGCTGTGTTCCCCAAAGTACA
RintHH_11820-4 (XhoI)	TGCCTCGAGACTCCTCATTTTCACTGTTG
