# Supplementary material for: Heterologous expression of genes from a cyanobacterial endosymbiont highlights substrate exchanges with its diatom host
Source: PNAS Nexus. 2023 Jun 27;2(6):pgad194. doi: 10.1093/pnasnexus/pgad194 (PMC10299089; doi:10.1093/pnasnexus/pgad194)
Supplement: pgad194_Supplementary_Data [file pgad194_supplementary_data.zip › PNASNEXUS-PNASNEXUS-2023-00139R-s01.pdf]

Supplementary Methods

Overexpression in *E.coli* of the RintHH_17430 (GlsR) protein. The synthetic gene bearing the NdeI/XhoI sites (excluding the N-terminal residues 1-40 of the protein signal sequence) was cloned in frame into NdeI/XhoI-digested pCMN28b (which is a vector derived from pET28b(+) in which the His tag has been replaced by a Strep-tag II sequence), to add a N-terminal Strep-tag II to the recombinant protein. The construct pMN13 containing the insert was corroborated by PCR analysis (Fig. S1B) and sequencing. This plasmid was transformed into *E. coli* DH5α and then subsequently into *E. coli* BL21(DE3) by electroporation. A pre-inoculum of this strain grown overnight in LB medium supplemented with 25 μg mL^{-1} of kanamycin (km) and 1% glucose was washed with LB medium and used to inoculate 1 L of LB medium and 25 μg mL^{-1} of km. The culture was incubated at 37 ºC up to an OD_{600nm} of 0.6, and protein expression was induced by addition of 1 mM isopropyl-β-D-1-thiogalactopyranoside (IPTG). After 2–3 h at 37 ºC, cells were collected by centrifugation (6,000 x *g*, 30 min, 4 ºC), washed with a buffer containing 50 mM Tris-HCl (pH 7.5), 200 mM NaCl and 10% glycerol, and centrifuged (6,000 x *g*, 30 min, 4 ºC). Cells were resuspended with buffer (50 mM Tris-HCl [pH 7.5], 200 mM NaCl, 10 % glycerol) at 5 mL g^{-1} of cells. The cell suspension was incubated with lysozyme (1 mg mL^{-1}) for 1 h at 4 ºC, and then protease inhibitor cocktail complete Mini EDTA-free (Roche) was added just before breakage of the cells by sonication (Digital Sonifier Branson, 4 x 30 s bursts). Soluble protein was then isolated by centrifugation (15,000 x *g*, 15 min, 4 ºC). Because the target protein was in the particulate fraction (Fig. S1C), cell extract was unfolded using a denaturation buffer (50 mM Tris-HCl [pH 7.5], 6 M guanidine hydrochloride, 10 mM $MgCl_2$, 10 μM EDTA, 10 mM DTT, 10 % glycerol) at 4 ºC overnight. Refolding was effected by dialysis against 1 L of refolding buffer (50 mM Tris-HCl [pH 7.5], 300 mM NaCl, 10 mMCl2, 1 mM DTT, 5 mM 2-mercaptoethanol, 20 % glycerol) containing from 6 M to 1 M urea. The buffer was changed every 1 h with a final dialysis of 30 min (from 2 M to 1 M of urea). Once removed from the dialysis tubing, protein was centrifuged (15,000 x *g*, 15 mins, 4 ºC) to remove any precipitated misfolded protein. RintHH_17430-Strep-tag II protein was purified from the supernatant using a 5-ml StrepTrap HP column from Sigma and 2.5 mM of desthiobiotin in binding buffer (100 mM Tris-HCl [pH 8], 150 mM NaCl). Samples obtained after purification were subjected to SDS-PAGE to confirm the presence of the protein (see Fig. S1D).

Overexpression in *E.coli* of the RintHH_3860 (InvB) protein. The PCR product from pMN7 (Fig. S4B) bearing the NdeI/XhoI sites was cloned in frame into NdeI/XhoI-digested pCMN28b (which is a vector derived from pET28b(+) in which the His tag has been replaced by a Strep-tag II sequence), to add a N-terminal Strep-tag II to the recombinant protein. The construct pMN11 containing the insert was corroborated by PCR analysis (Fig. S5C) and sequencing. This plasmid was transformed into *E. coli* DH5α and then subsequently into *E. coli* BL21(DE3) by electroporation. A pre-inoculum of this

strain grown overnight in LB medium supplemented with 25 μg mL^{-1} of km and 1% glucose was washed with LB medium and used to inoculate 1 L of LB medium and 25 μg mL^{-1} of km. The culture was incubated at 37 ºC up to an OD_{600nm} of 0.6, and protein expression was induced by addition of 1 mM isopropyl-β-D-1-thiogalactopyranoside (IPTG). After 2–3 h at 37 ºC, cells were collected by centrifugation (6,000 x *g*, 30 min, 4 ºC), washed with a buffer containing 20 mM HEPES pH 6.8, 100 mM NaCl, 1 mM EDTA, 5 mM β-mercaptoethanol, 5% glycerol, and centrifuged (6,000 x *g*, 30 min, 4 ºC). Cells were resuspended with buffer (20 mM HEPES pH 6.8, 100 mM NaCl, 1 mM EDTA, 5 mM β-mercaptoethanol, 5% glycerol) at 5 mL g^{-1} of cells. The cell suspension was incubated with lysozyme (1 mg mL^{-1}) for 1 h at 4 ºC, and then protease inhibitor cocktail complete Mini EDTA-free (Roche) was added just before breakage of the cells by sonication (Digital Sonifier Branson, 4x30 s bursts). The resulting material was subjected to centrifugation (15,000 x *g*, 15 min, 4 ºC) and the target protein was found in particulate fraction as was shown in SDS-PAGE (Fig. S5D). We then used cell-free extract for invertase analysis.

Overexpression in *E.coli* of RintHH_12770, RintHH_7180 and RintHH_11820. The synthetic genes RintHH_12770 and RintHH_7180 provided in plasmid pUCIDT by Integrated DNA Technologies, Inc. (IDT) were amplified by PCR with the pairs of primers RintHH_12770-3/4 and RintHH_7180-3/4, respectively. These pairs of primers include NdeI and XhoI restriction sites. The PCR product for RintHH_12770 (excluding the N-terminal residues 1-21 of the protein signal sequence) and for RintHH_7180 (excluding the N-terminal residues 1-15 of the protein signal sequence) were digested with NdeI and XhoI enzymes, and cloned in frame into the NdeI/XhoI-digested pET28b+ to add an N-terminal His-tag to the recombinant proteins. The synthetic gene RintHH_11820 was amplified by PCR (excluding the N-terminal residues 1-23 of the protein signal sequence) with the pair of primers RintHH_11820-3/4, which include a NcoI and XhoI restriction sites, respectively. The PCR product, after digestion with these enzymes, was cloned in frame into the NcoI/XhoI-digested pET28b+ to add a C-terminal His-tag to the recombinant protein. All the constructs containing the corresponding inserts were corroborated by PCR analysis (Fig. S8B, S9B and S7B) and sequencing. These plasmids were transformed into *E. coli* DH5α and then subsequently into *E. coli* BL21(DE3) by electroporation. RintHH_12770, RintHH_7180 and RintHH_12690 proteins were overproduced in E. coli BL21 (DE3) cells. After growth at 37 ºC up to an OD_{600nm} of 0.6, cells were induced with the addition of 1 mM isopropyl-β-D-1-thiogalactopyranoside (IPTG) and shaken at 250 rpm for 3 hours before harvesting by centrifugation (5,000 x *g*, 30 mins, 4 ºC). Pelleted cells were washed with a buffer containing 100 mM Tris-HCl (pH 7.0) and 150 mM NaCl and centrifuged (7,000 x g, 10 min, 4 ºC). Cells were resuspended with a buffer containing 100 mM Tris-HCl [pH 7.0], 150 mM NaCl, and 10 % glycerol at 5 mL g^{-1} of cells. The cell suspension was incubated with lysozyme (1 mg ml^{-1}) for 1 h at 4 ºC, and then a protease inhibitor cocktail complete Mini EDTA-free (Roche) was added just before breakage of the cells by

sonication (Digital Sonifier Branson, 4 x 30 s bursts). Soluble fractions were isolated from particulate fractions by centrifugation (15.000 x g, 25 min 4 ºC) to produce cell-free extracts. Because the target proteins were in the particulate fractions (Fig. S8C, S9C and S7C), proteins were solubilized using a buffer containing 50 mM Tris-HCl [pH 8.0], 6 M guanidine hydrochloride, 10 % glycerol, 10 mM DTT and 1 mM EDTA at 4 ºC overnight. The solubilized extracts were cleared by centrifugation at 15,000 x g, 30 min, 4 ºC to remove the membrane particles, and then filtered using a filter with a pore diameter of 0.22 μm. Then, extracts were passed through a 1-mL HisTrap-FF column (GE healthcare, UK) following the instructions of the manufacturer, and the material retained was washed with buffer A (50 mM Tris-HCl [pH 8.0], 300 mM NaCl, 10% glycerol, 5 M Urea, 20 mM imidazole, 1 mM DTT, 5 mM β-mercaptoethanol) and were eluted with buffer B (buffer A supplemented with increasing concentrations of imidazole). Samples obtained after purification were subjected to SDS-PAGE to confirm the presence of the protein (see Fig. S8D, S9D and S7D). The purified proteins were refolding by serial dialysis against 1 L of buffer C (50 mM Tris-HCl pH [8.0], 200 mM KCl, 20% glycerol, 1 mM DTT, 5 mM β-mercaptoethanol) containing decreasing amounts of urea (1 h with 5 M urea, 1 h with 4 M urea, 1 h with 3 M urea, 1.5 h with 2 Murea, and 1 h with 1 M urea), and finally against buffer D (50 mM Tris-HCl [pH 8.0], 200 mM KCl, 20% glycerol). Following the dialysis, the preparations were cleared by centrifugation at 15,000 x g, 30 min, 4 ºC. Protein concentration was estimated by a dye-binding assay (Bio-Rad).

Testing specificity of TaqMan qPCR assays (*in silico*). To ensure the RT-qPCR assays were specific to ReuHH01, BLASTn analyses with the oligonucleotide sequences were performed. The databases used for the BLASTn analyses consist of genomes downloaded from the National Center for Biotechnology Information (NCBI) database Refseq (1–3) and GenBank (4), and additionally include the latest version of Genome Taxonomy Database (GTDB; v. R07-RS207) (5) and subsequently annotated using Prokka (v. 1.14.6) (6). These databases include: (I) representative bacterial and archaeal genomes classified in GTDB (198 569 917 sequences) and (II) representative cyanobacteria genomes classified in GTDB (9 680 513 sequences). In accordance with GTDB taxonomy, only one representative genome is available per species and all genomes are available in NCBI. Since the TaqMan assay requires that all primers and the probe bind in order for the reaction to occur, alignment results were filtered by only selecting alignments which used the full sequence of the primers and probe as queries. Therefore, only genomes for which all three oligonucleotides were significantly aligned should be considered for potential cross-reactivity, i.e., non-specificity. BLASTn analyses against Database I did not indicate cross-reactivity for any of the assays (Fig. S10A). Alignments against Database II indicated a 91% sequence similarity in the Forward primer and 96% sequence similarity for the probe and reverse primer for the *potD* qPCR assay with the closely related *R. euintracellularis* HM01 (Fig. S10B). These sequence similarities are equivalent to 2 and 1 mismatches, respectively (Fig

S11). Of importance here is given the location of the 2 mismatches in the forward primer on the 3′ end, and the additional mismatches on both the probe and reverse primer on the 5' ends, should result in a less than optimal efficiency in the qPCR reaction. Thus, the expected cross-reactivity with *R. euintracellularis* HM01 should be low.

Gene expression of *glsR, invB, natF, potD*, and *secA* in environmental samples. Total RNA was extracted using the Qiagen RNA easy kit (Qiagen) as described previously and re-eluted in 30 µl (47). Two µl of extracted RNA was reverse transcribed to cDNA using the SuperScript™ III First strand synthesis system (Invitrogen), according to manufacturer's instructions. A second set of reactions were run without reverse transcriptase (RT) and served as no-RT controls. A third set of reactions included water as a template in reactions with and without RT. After RT (and no-RT) reactions, 2 µl of cDNA was used as template in qPCR reactions as previously described (47). All qPCRs were conducted in a StepOnePlus™ Real-Time PCR System (Applied Biosystems) using the default conditions for the reaction parameters (50°C for 2 min, 95°C for 10 min, 45 cycles at 95°C for 15s and 60°C for 2 min). The target-specific TaqMAN (Applied Biosystems) oligonucleotides were designed as indicated in Suppl. Table S2 and synthesized by IDT. Each probe was 5'labelled with a fluorescent reporter FAM (6-carboxyfluoresceom) and 3' labelled with TAMRA (6-carboxytetramethulrhodamine) as a quenching dye. All qPCRs contained 12.5 µl of 2X TaqMan buffer (Applied Biosystems), 10 µl of molecular grade water, 1.0 µl each of forward and reverse primers (10 µM), 0.5 µl probe (10 µM), and 2 µl of cDNA template or separately 2 µl of water for no template controls and 2 µl of standards. Duplicate standard curves of synthesized gBlocks (IDT) were made in a dilution series ranging 10^8 to 10^0 gene copies per reaction and run in parallel with all samples. Samples were run in triplicate for cDNA and gene copies calculated from the mean C_t value of the three replicates and the standard curve for the appropriate primer and probe set. Samples which had 1 or 2 of the 3 replicates detected are reported as detected, not quantifiable in Suppl. Table S3; the limit of detection for the qPCR assays is 1-10 copies.

References

1. Altschul, S. Gapped BLAST and PSI-BLAST: a new generation of protein database search programs. *Nucleic Acids Research* **25**, 3389–3402 (1997).

2. Brister, J.R., Ako-adjei, D., Bao, Y. & Blinkova, O. NCBI Viral Genomes Resource. *Nucleic Acids Res* **43**, D571–D577 (2015).

3. O'Leary, N.A., Wright, M.W., Brister, J.R., Ciufo, S., Haddad, D., McVeigh, R. *et al.* Reference sequence (RefSeq) database at NCBI: current status, taxonomic expansion, and functional annotation. *Nucleic Acids Res* **44**, D733–D745 (2016).

4. Benson, D.A., Cavenaugh, M., Clark, K., Karsch-Mizrachi, I., Lipman, D.J., Ostell, J. *et al.*

GenBank. *Nucleic Acids Res* **45**, D37–D42 (2017).

5. Parks, D. H., Chuvochina, M., Waite, D.W., Rinke, C., Skarshewski, A., Chaumeil, P-A. *et al.*, A standardized bacterial taxonomy based on genome phylogeny substantially revises the tree of life. *Nat Biotechnol* **36**, 996–1004 (2018).

6. Seemann, T. Prokka: rapid prokaryotic genome annotation. *Bioinformatics* **30**, 2068–2069 (2014).
